# An Anatomic Characterization of the Midbrain Near Response Neurons in the Macaque Monkey

**DOI:** 10.1167/iovs.17-23737

**Published:** 2018-03

**Authors:** Paul J. May, Susan Warren, Paul D. R. Gamlin, Isabelle Billig

**Affiliations:** 1 Department of Neurobiology & Anatomical Sciences, University of Mississippi Medical Center, Jackson, Mississippi, United States; 2 Department of Ophthalmology, University of Mississippi Medical Center, Jackson, Mississippi, United States; 3 Department of Neurology, University of Mississippi Medical Center, Jackson, Mississippi, United States; 4 Department of Ophthalmology, University of Alabama at Birmingham, Birmingham, Alabama, United States; 5 Systems Neuroscience Institute, University of Pittsburgh, Pittsburgh, Pennsylvania, United States

**Keywords:** Edinger-Westphal, ciliary ganglion, lens accommodation, vergence, supraoculomotor area

## Abstract

**Purpose:**

These experiments were designed to reveal the location of the premotor neurons that have previously been designated physiologically as the midbrain near response cells controlling vergence, lens accommodation, and pupillary constriction in response to target distance.

**Methods:**

To identify this population, the fixed N2c strain of rabies virus was injected into the ciliary body of seven *Macaca fascicularis* monkeys. The virus was trans-synaptically transported to the brain. Following a 58- to 76-hour survival, animals were perfused with formalin fixative. After frozen sectioning, tissue was reacted to reveal the location of the infected populations by use of a monoclonal anti-rabies antibody. Another series of sections was processed to determine which of the rabies-positive cells were cholinergic motoneurons by use of an antibody to choline acetyl transferase.

**Results:**

At earlier time points, only cholinergic cells in the preganglionic Edinger-Westphal nucleus ipsilateral to the injection were labeled. At later time points, an additional population of noncholinergic, premotor cells was present. These were most numerous at the caudal end of the supraoculomotor area, where they formed a bilateral band, oriented mediolaterally immediately above the oculomotor nucleus. Rostral to this, a smaller bilateral population was located near the midline within the supraoculomotor area.

**Conclusions:**

Most lens preganglionic motoneurons are multipolar cells making up a continuous column within the Edinger-Westphal nucleus. A population of premotor cells that likely represents the midbrain near response cells is located in the supraoculomotor area. These cells are bilaterally distributed relative to the eye they control, and are most numerous caudally.

In addition to the well-studied gaze changes made to targets in the same plane, the eyes often move between targets that are at different distances from the viewer. These gaze changes involve disjunctive, as opposed to conjugate, movements of the eyes to foveate the target, and in addition they involve changes in lens accommodation and pupil size to focus the target on the retina (the so-called near triad). Mays^1^ and Judge and Cumming^2^ were the first to reveal the central mechanisms underlying these types of gaze changes. These investigators described a set of neurons that lay in the vicinity of the oculomotor nucleus (III), and had firing rates that were correlated with either vergence angle or lens accommodation, or both. These neurons were termed the midbrain near response cells. Further studies described vergence velocity neurons, which had a burst of activity for vergence movements, in the same general region.^3^ By means of antidromic activation, these midbrain near response cells were shown to project to medial rectus motoneurons to generate vergence eye movements.^4^ Based on recordings from preganglionic motoneurons of the Edinger-Westphal nucleus (EWpg) during ocular accommodation, it was suggested that these cells also receive input from midbrain near response neurons.^5^ While some authorities have suggested that disjunctive saccades are produced by the activity of eye-specific neurons in the saccade circuitry,^6–8^ it remains clear that the midbrain near response population is an important component of the brainstem circuitry that directs near triad behaviors for eye movements to targets in three-dimensional space.

Despite the importance of this population, they have not been described anatomically. They are located so near to their axonal targets, the medial rectus motoneurons in III and the EWpg, that it is difficult to approach them with conventional neuroanatomic tracer techniques. Retrograde trans-synaptic tracer techniques have been applied to the medial rectus muscle of the guinea pig.^9^ This study revealed labeled cells in the supraoculomotor area (SOA), dorsal to III, and in the midbrain reticular formation, ventrolateral to the medial longitudinal fasciculus (MLF). However, there is no way to establish whether these populations provide conjugate or disconjugate signals to the medial rectus motoneurons. The lack of an anatomic handle on this population has made it difficult to identify their inputs and so further examine the circuitry that controls the important visuomotor capability of controlling the components of the near response.

To fill this void in our knowledge, we have adopted a different trans-synaptic approach. In the experiments described here, we injected rabies virus into the ciliary body (Fig. 1). We reasoned that the viral particles would be taken up by the myoneuronal junctions of postganglionic motoneurons whose somata are located in the ciliary ganglion and whose axons supply the ciliary muscle to produce lens accommodation. After multiplying in these first-order cells, the virus would pass across the synapses in the ganglion to label the preganglionic fibers and, after retrograde transport, they would come to reside in the second-order, lens accommodation-related preganglionic motoneurons of the EWpg. This subpopulation has not been previously described, and there is argument over the organization of the EWpg, with some authorities describing it as a unitary nucleus consisting of a single, rostrocaudally running column^10^ and other authorities claiming it is divided into discrete subnuclei.^11^ After another round of replication, the viral particles would again cross the synapses to invade the axons of third-order cells supplying input to these motoneurons. Since midbrain near response cells are the third-order premotor neurons controlling lens accommodation, they would become labeled at this point (Fig. 1).

**Figure 1:**
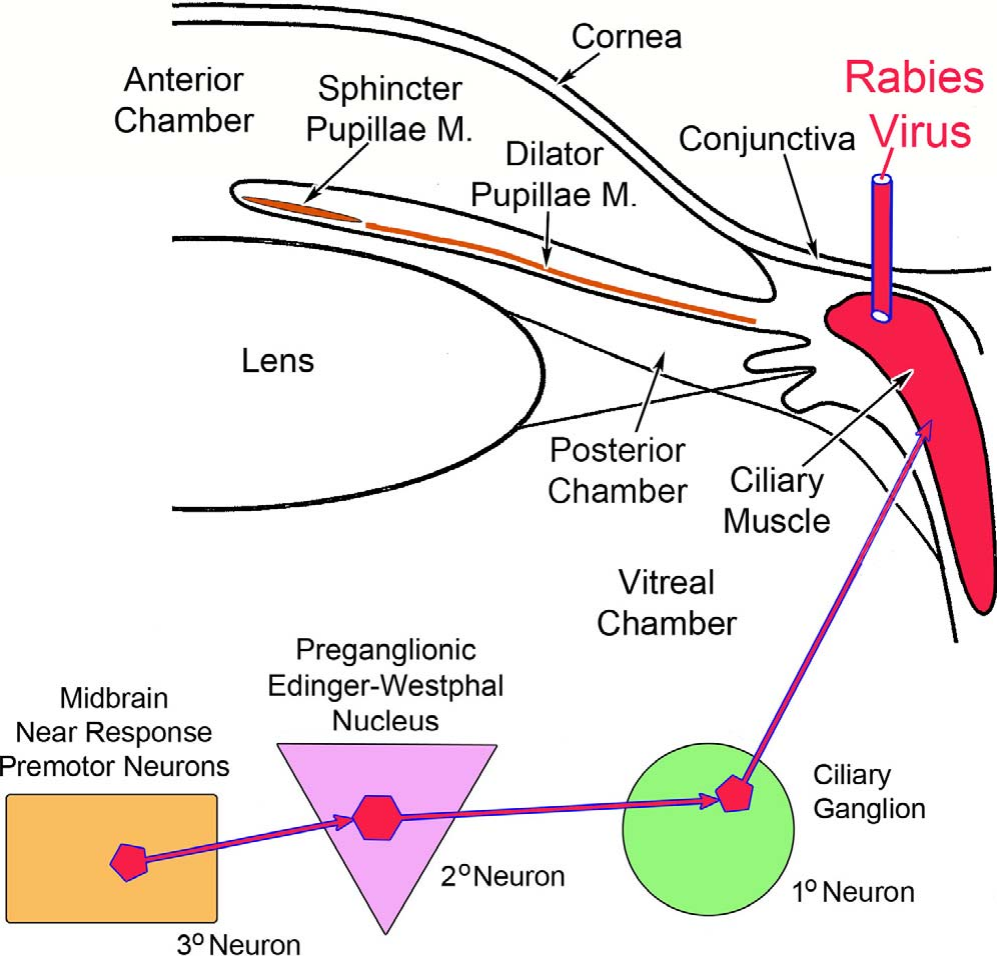
Schematic of the experimental model. Rabies virus (red) injected into the ciliary body was taken up by the axons of first-order postganglionic motoneurons in the ciliary ganglion that supply the ciliary muscle. It was then transferred to second-order preganglionic motoneurons in the Edinger-Westphal nucleus. Finally, it infected the third-order premotor neurons supplying the preganglionic motoneurons.

## Methods

A total of seven *Macaca fascicularis* monkeys of both sexes were used in this study. All animal procedures were approved by the University of Pittsburgh Institutional Animal Care and Use Committee, and they were carried out in line with tenets of the Guide for Care and Use of Animals and the ARVO Statement for the Use of Animals in Ophthalmic and Vision Research. All surgeries took place at the University of Pittsburgh in the Center for Neuroanatomy and Neurotropic Viruses directed by Peter L. Strick, PhD. Animals were sedated with ketamine HCl (10–15 mg/kg, intramuscularly [IM]), intubated, and then anesthetized with isoflurane (3%). Their heads were stabilized in a stereotaxic apparatus (David Kopf Instruments, Tujunga, CA, USA). They were then prepped for sterile surgery. Proparacaine ophthalmic drops were placed on the left cornea. A fixed N2c strain of the rabies virus (1 × 10^9^ plaque forming units [pfu]/mL for 66- and 72-hour cases, 5 × 10^9^ pfu/mL for 58- and 76-hour cases) was drawn up into a 100-μm Hamilton syringe equipped with a 25-gauge needle. We found that this strain travels in autonomic motoneurons, in contradistinction to other strains.^12^ The 2.0-mm needle tip was inserted by hand, just lateral to the corneoscleral junction, and was driven just under the surface. Between 130 and 170 μL of the viral solution was spread between 12 and 16 sites along the ventral, lateral, and dorsal perimeter of the cornea. To allow spread of the injected virus into the appropriate tissue, the needle was retained in place for 1 to 2 minutes. The cornea was rinsed with saline following the injection to reduce the possibility of contaminating other tissues.

The animal was supplied with buprenorphine HCl (0.01 mg/kg, IM) as a postoperative analgesic. The monkeys survived for 58 (*n* = 1), 66 (*n* = 2), 72 (*n* = 2), or 76 (*n* = 2) hours. They were then sedated with ketamine HCl (25 mg/kg, IM) and deeply anesthetized with sodium pentobarbital (40 mg/kg, intraperitoneally [IP]). Once they were entirely unresponsive, they were transcardially perfused with a rinse of phosphate-buffered saline (0.1 M, pH 7.4) followed by a 10% formalin fixative in 0.1 M, pH 7.4 phosphate buffer (PB). This was followed by perfusion with additional buffered fixative solution that contained 10% glycerol to prepare the brain for cryosectioning. The brains were blocked in the frontal plane, extracted, and placed in the same buffered formalin and glycerol solution. They were postfixed in this solution for 7 to 10 days at 4°C. In addition, the cervical and thoracic spinal cord, as well as the ciliary, pterygopalatine, superior cervical, and trigeminal ganglia, were extracted and placed in the same solution.

The brain, spinal cord, and ganglia were frozen and sectioned at 50 μm on a sliding microtome (American Optical, Buffalo, NY, USA). The sections were collected in 0.1 M, pH 7.4 phosphate Trizma buffer (Fisher Scientific, Houston, TX, USA) with 0.05% sodium azide (PTA) and stored at 4°C. Sections from a 1 in 10 series were reacted to reveal the location of the virus. First, background peroxidase activity was quenched by treating the sections with 0.3% hydrogen peroxide solution. Following PTA rinses, the sections were then treated to suppress nonspecific antibody reactions by immersion in a 1.5% normal horse serum solution of PTA. This solution also contained 0.5% Triton X-100 to increase tissue penetrance. To identify the rabies-infected cells, the sections were incubated in a mouse monoclonal antibody to rabies virus (diluted 1:1000 in PTA) for 2 days at 4°C with gentle agitation. (This antibody was a generous gift of Matthias Schnell and has been designated clone 31G10.) Following rinses in 0.1 M, pH 7.4 phosphate Trizma buffer, the antibody was tagged using a Vector ABC kit (Vector Laboratories, Burlingame, CA, USA). Specifically, the sections were treated with biotinylated anti-mouse IgG, and then with horseradish peroxidase–conjugated avidin. This was followed by reaction with a 0.05% diaminobenzidine (DAB) solution in 0.1 M, pH 7.2 PB with 0.016% hydrogen peroxide to produce a brown chromagen. Following rinsing with PB, the sections were mounted, cleared, and coverslipped. An adjacent 1 in 10 series was mounted on slides, then stained with cresyl violet before coverslipping. At least one other 1 in 10 series underwent the immunolocalization procedures and was counterstained with cresyl violet. We sampled tissue with a maximum of 250-μm spacing.

To establish which of the neurons infected with the trans-synaptically transported rabies virus represented preganglionic motoneurons, we employed a dual-fluorescence procedure. As before, a 1 in 10 series of sections were treated with normal horse serum and Triton X-100. They were then placed in the mouse monoclonal, anti-rabies antibody solution for 2 days at 4°C. After rinsing in PTA they were incubated in Cy2-conjugated donkey anti-mouse IgG (Jackson ImmunoResearch, West Grove, PA, USA) (1:500 in PB) with gentle agitation at room temperature for 6 hours. Next, they were rinsed in PB and placed in the second antibody solution: goat anti-choline acetyl transferase (ChAT) polyclonal antibody (Millipore AB144P; Millipore Sigma, St. Louis, MO, USA). This antibody was diluted 1:480 in 0.1 M, pH 7.2 PB with 1% bovine serum albumin (BSA) and 0.3% Triton X-100. The sections were incubated for 2 days at 4°C with gentle agitation. The goat antibody was then tagged with biotinylated donkey anti-goat IgG (Jackson ImmunoResearch), diluted 1:500 in 1% BSA, 0.3% Triton X-100 in PB for 2 hours with agitation at room temperature. Following PB rinses, the sections were incubated in Cy3-conjugated streptavidin (Jackson ImmunoResearch) diluted 1:200 in 1% BSA, 0.3% Triton X-100 in PB for 2 hours with agitation at room temperature. They went through final rinses and were mounted on slides, dehydrated, and cleared. They were then coverslipped with nonfluorescing medium (Cytoseal 60; VWR Scientific, Atlanta, GA, USA).

Analysis and charting of the DAB-labeled material were accomplished with an Olympus BH-2 microscope (Olympus, Tokyo, Japan) that was equipped with a drawing tube. Low-magnification drawings of sections were made using a Wild M-8 stereoscope (Leica Microsystems, Buffalo Grove, IL, USA). Images of the DAB and immunohistochemically stained cells were taken with a Nikon Eclipse E600 fluorescence photomicroscope (Nikon Instruments, Mellville, NY, USA) equipped with a Nikon DS-ri1 digital camera. Images were adjusted in Adobe Photoshop (Adobe Systems, San Jose, CA, USA) to resemble the appearance of the section as viewed with the eyes. Chartings of the fluorescent cells were made by using prints of these images overlain with tracing paper.

## Results

**Figure 2:**
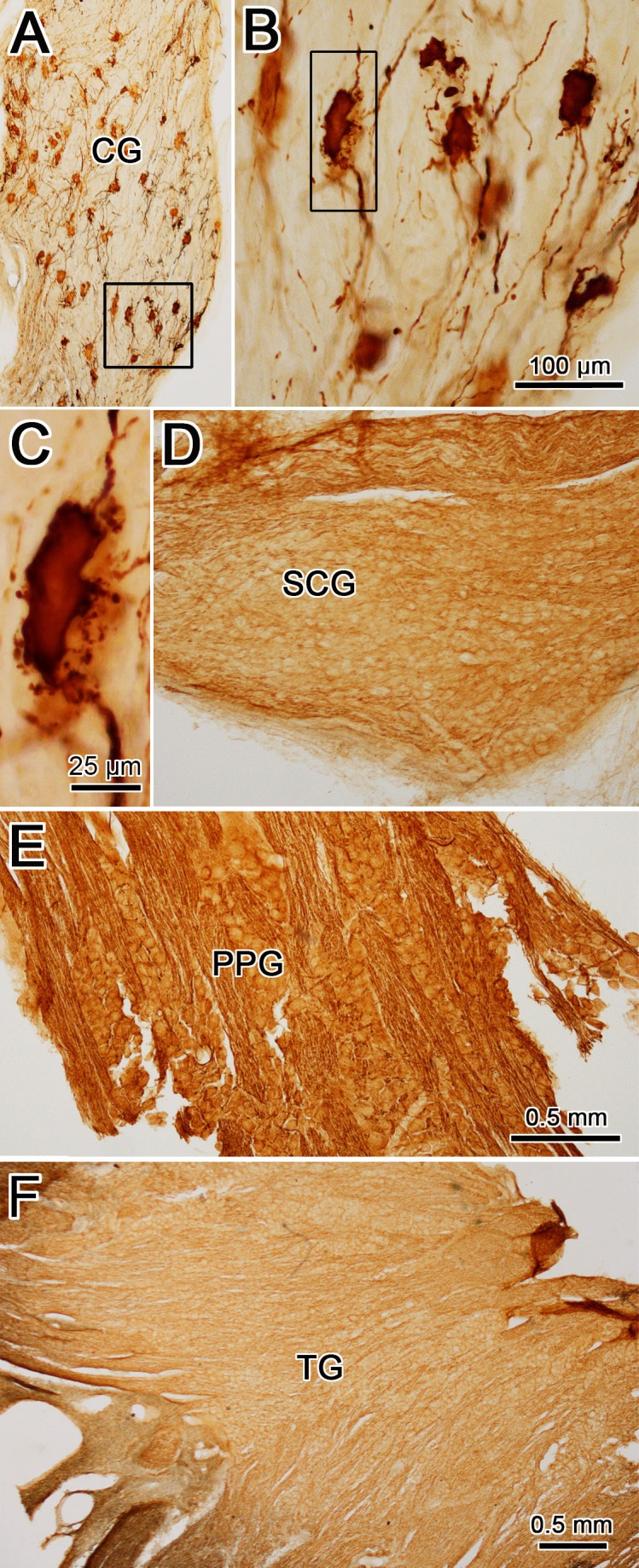
Examples of labeling of the cranial ganglia. (A) Following injection of the ciliary body with rabies virus, brown labeled cells were evident throughout the ciliary ganglion (CG). (Box indicates area shown in [B].) (B, C) Higher-magnification views show the largely unbranched dendrites and small perisomatic processes of the labeled ciliary ganglion cells (box in [B] indicates area shown in [C]). No labeled ganglion cells are apparent in the superior cervical ganglion (SCG) (D), pterygopalatine ganglion (PPG) (E), or trigeminal ganglion (TG) (F). Scale bar for A and D is shown in F.

Several ganglia, including the ciliary ganglion (CG), superior cervical ganglion (SCG), pterygopalatine ganglion (PPG), and trigeminal ganglion (TG), were analyzed to ensure that uptake of the rabies virus was constrained to postganglionic motoneurons supplying the ciliary muscle. Examples are provided in Figure 2. Numerous labeled cells were found in the CG (Fig. 2A). Closer examination of these motoneurons (Fig. 2B) revealed that they had just a few, long, sparsely branching dendrites that extended within the CG. They also displayed many small perisomatic processes that appear as a halo around the soma (Fig. 2C). No labeled cells were observed in the SCG (Fig. 2D), PPG (Fig. 2E), or TG (Fig. 2F).

**Figure 3:**
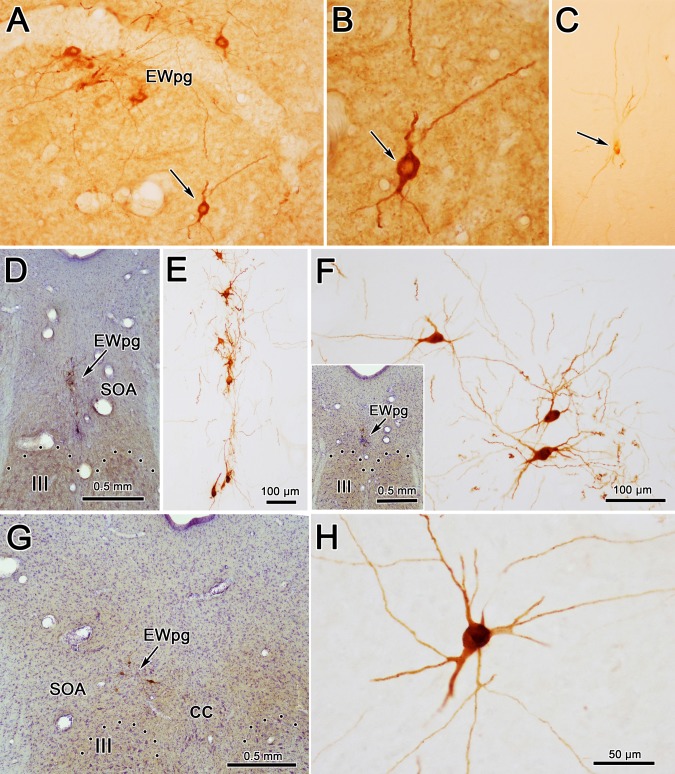
Morphology of labeled neurons in the ipsilateral preganglionic Edinger-Westphal nucleus (EWpg) at 58 hours (A–C) and 66 hours (D–H) after rabies injections into the ciliary body. The rare, densely labeled (brown) cell indicated by the arrow in (A) is shown at higher magnification in (B). However, at 58 hours, most cells were quite lightly labeled, like the cell indicated by the arrow in (C). Counterstained sections (D, insert F, G) show examples from rostral pole, middle, and caudal oculomotor nucleus (III) levels. The brown dendrites of the intensely stained rabies-positive cells are easily seen in the adjacent noncounterstained sections. They tend to be dorsoventrally oriented, rostrally (E), but show no orientation, caudally (F, H). Dotted line indicates dorsal border of III. Scale bar for A and C is shown in F.

**Figure 4:**
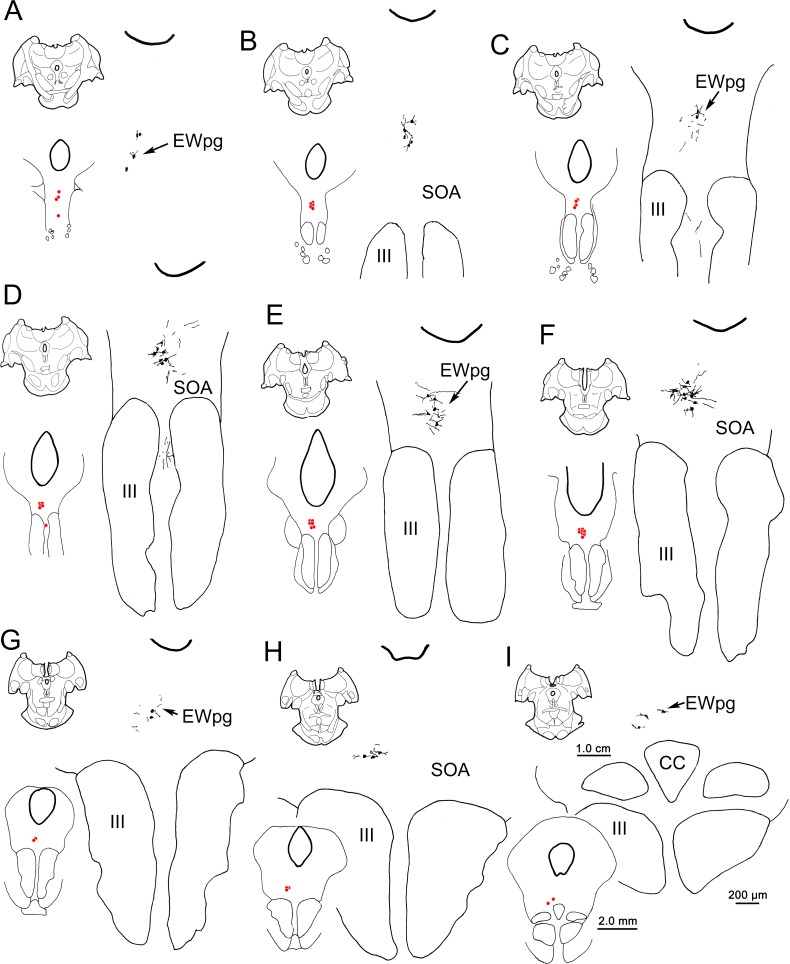
The distribution of labeled neurons in the ipsilateral preganglionic Edinger-Westphal nucleus (EWpg) at 58 hours after the rabies injection of the left ciliary body. Labeled cells were present throughout the EWpg (A–I). For each level, the section is shown, as well as a medium-magnification charting of the labeled cells as red dots. The high-magnification view also shows the distribution of labeled dendrites. Cell labeling stretched rostral to the oculomotor nucleus (III) (A) and caudally, extended to the level of the caudal central subdivision (CC). The labeled dendrites extended into the supraoculomotor area (SOA) immediately outside the EWpg. Scale bars for the three illustrated magnifications shown in (I).

The appearance of the labeled cells in the midbrain following a 58-hour survival is shown in Figures 3A through 3C. Nearly all of these cells were fairly lightly labeled, like the example shown in Figure 3C. By extending the incubation and reaction times, we were able to produce denser labeling, albeit with a concomitant increase in background staining. A rare, heavily labeled example of these medium-sized multipolar neurons is shown in Figure 3B. The location of these labeled neurons is charted in Figure 4. These cells formed a rostrocaudally running column located dorsal to III (Figs. 4B–I). This column extended slightly rostral to III (Fig. 4A). It moved slightly laterally at its caudal end and it terminated shortly after the appearance of the caudal central subdivision (CC) (Figs. 4H, 4I). Based on their location, most of these labeled cells were located in the area of the EWpg. However, a few were observed in between the rostral poles of the two oculomotor nuclei (Fig. 4D). These may represent a caudal continuation of the EWpg population that wraps around the rostral pole of III (Fig. 4A).

**Figure 5:**
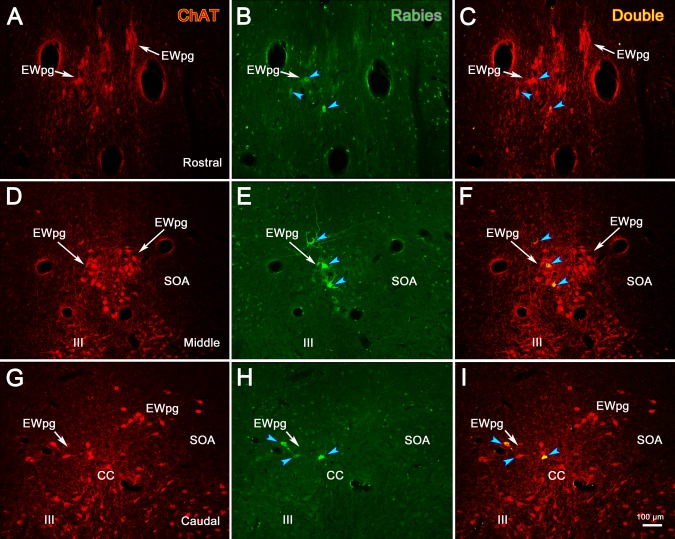
In animals that survived 58 hours after the injection of rabies into the left ciliary body, all the labeled cells in the Edinger-Westphal nucleus (EWpg) are cholinergic motoneurons. Examples from rostral to the oculomotor nucleus (III) (A–C), the middle of III (D–F), and the caudal end of III, where the caudal central subdivision (CC) is present (G–I), are shown. Immunohistochemistry for choline acetyltransferase (ChAT) caused preganglionic motoneurons and somatic motoneurons in III to fluoresce red (A, D, G). In contrast, the rabies-positive cells fluoresced green (B, E, H). Examples of the rabies-positive cells that were also ChAT-positive are indicated by blue arrowheads in the combined images (C, F, I). The most heavily labeled of these appear yellow. The scale bar for all plates is found in (I).

**Figure 6:**
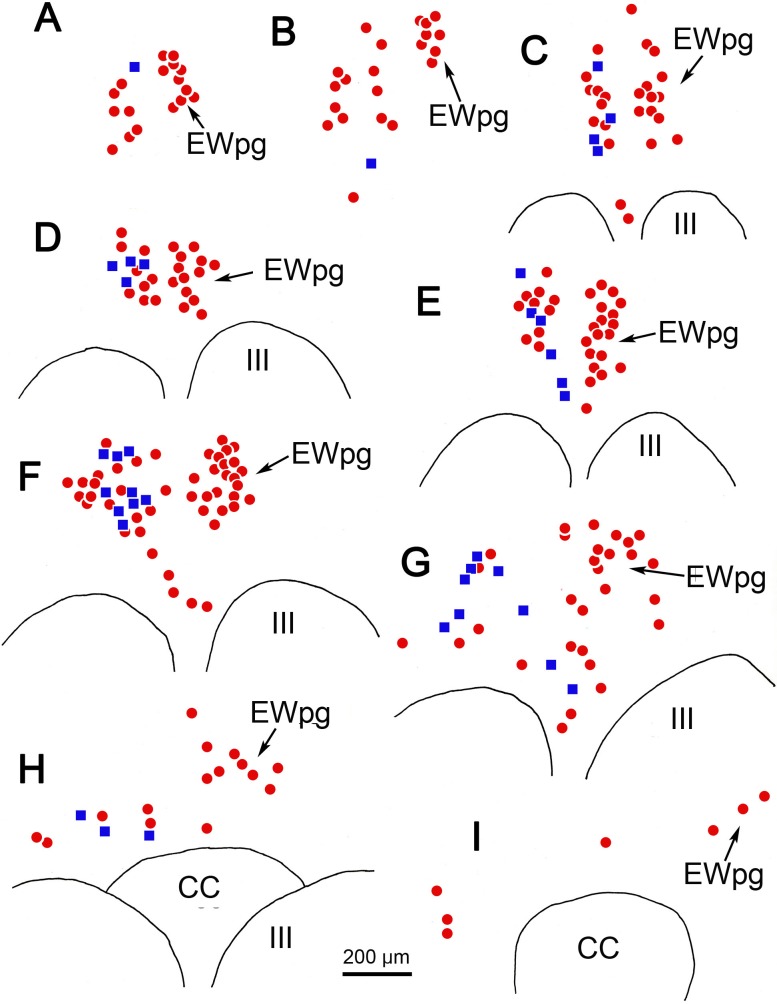
Distribution of rabies-positive and choline acetyltransferase (ChAT)-positive cells rostral to (A, B) and above (C–I) the ipsilateral oculomotor nucleus (III) and caudal central subdivision (CC) at 58 hours after a rabies injection into the left ciliary body. The ChAT-positive but rabies-negative cells are indicated by red dots. The ChAT-positive, rabies-positive cells are indicated by blue squares. These double-labeled cells were present throughout the entire rostrocaudal column (A–I).

Due to this organization, we posited that these cells represented preganglionic motoneurons. To test this, we utilized dual-fluorescence immunohistochemical techniques targeting rabies and ChAT. In Figure 5, the cholinergic motoneurons were labeled red using anti-ChAT antibody (Figs. 5A, 5D, 5G); the infected cells were labeled green using anti-rabies antibody (Figs. 5B, 5E, 5H); and double-labeled cells that contain both ChAT and rabies virus appeared yellow when the images were combined (Figs. 5C, 5F, 5I). As shown from these sections taken rostral to III (Figs. 5A–C), at the middle of the rostrocaudal extent of III (Figs. 5D–F), and at the level of the CC (Figs. 5G–I), all the rabies-positive cells were also ChAT-positive. This organization is further illustrated in the chartings presented in Figure 6. The cholinergic, preganglionic motoneurons (red dots) are largely confined to a column of cells that lies dorsal to III. This group forms a somewhat loose cluster termed the EWpg (Figs. 6C–I), and it extends rostral to the ChAT-positive cells in III, as part of the anteromedian nucleus (Figs. 6A, 6B). There are scattered ChAT-positive cells in the area between EWpg and III. All the rabies-positive cells in this case were ChAT-positive, indicating that they were all cholinergic motoneurons (Figs. 6A–H, blue squares). A few of the rabies-positive cells were located in the SOA, ventral to EWpg. These were also ChAT-positive (Figs. 6E, 6G, blue squares).

**Figure 7:**
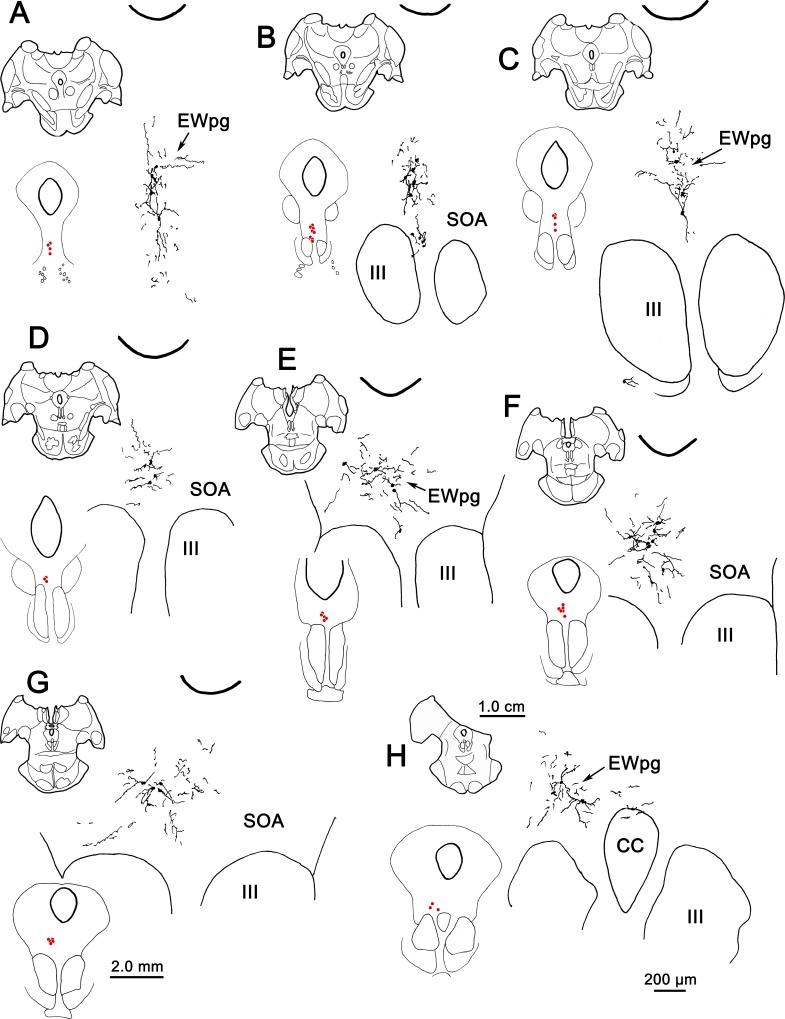
The distribution of labeled neurons in the ipsilateral preganglionic Edinger-Westphal nucleus (EWpg) at 66 hours after an injection of rabies into the left ciliary body. Labeled cells were present throughout the ipsilateral EWpg (A–H). For each level, the section is shown, as well as a medium-magnification charting of the labeled cells as red dots. The high-magnification views show the distribution of labeled dendrites, which extend well into the supraoculomotor area (SOA) and sometimes cross the midline. Cell labeling stretched rostral to the oculomotor nucleus (III) (A) and extended to the level of the caudal central subdivision (CC) (H).

When the survival time was extended to 66 hours, the number of cells in the EWpg was increased and the entire dendritic field of these cells was now evident (Figs. 3D–H). Variations in the organization of these dendritic fields are illustrated in Figures 3 and 7. Rostral to III (Fig. 7A) and above the rostral pole of the nucleus (Figs. 3D, 3E, 7B, 7C), the labeled cells formed a vertical column and their dendrites were largely oriented within this column. Further caudally (Figs. 3F, 7D–F), the labeled multipolar cells showed no apparent dendritic orientation and their dendrites extended out into the SOA and across the midline. At the caudal end of their distribution (Figs. 3G, 3H, 7G, 7H), the labeled cells moved slightly laterally as the CC appeared (Fig. 7H), and the multipolar neurons continued to extend their dendrites well into the surrounding SOA. Note that the dendrites never extend down into III. In this animal, dual-fluorescence immunohistochemistry revealed that one or two lightly labeled, rabies-positive, noncholinergic neurons were present in SOA per series. In the second 66-hour animal (not illustrated), more of these cells were present, indicating that the labeling of third-order premotor neurons had begun. However, we will illustrate this third-order pattern with longer-survival-period animals where the morphology and distribution of the neurons are clearer.

**Figure 8:**
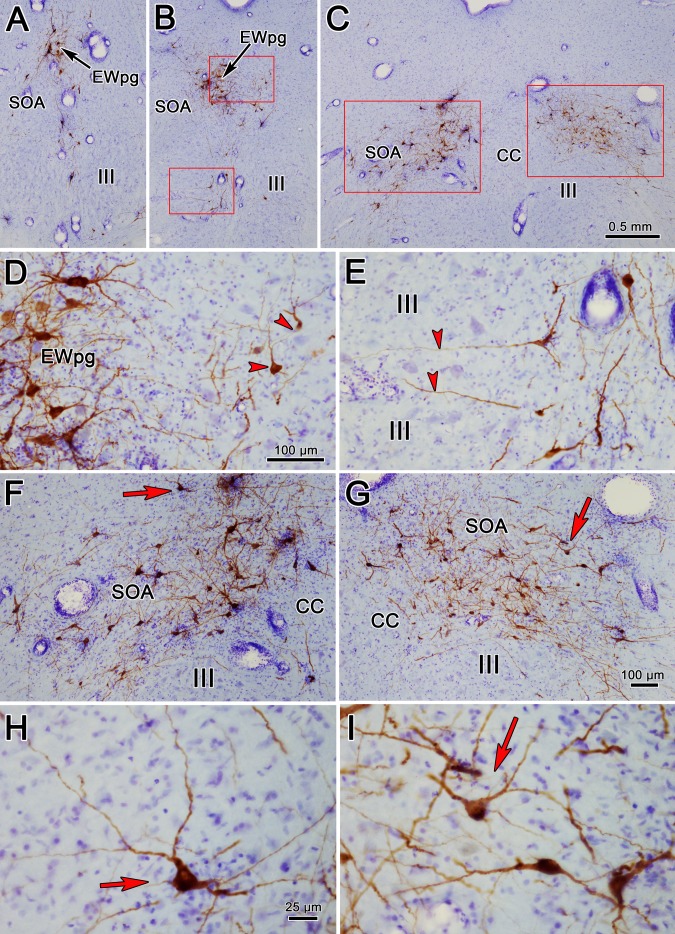
The morphology of rabies-positive neurons located above the oculomotor nucleus (III) at 76 hours after an injection of rabies into the left ciliary body (A–C). Rostral (A), middle (B), and caudal levels (C) through the labeled population are shown, with red boxes indicating the areas shown at higher magnification in (D–I). (D) The region indicated by the upper box in (B) shows intensely labeled (brown) cells in the ipsilateral preganglionic Edinger-Westphal nucleus (EWpg), as well as cells in the contralateral nucleus (red arrowheads). (E) The region indicated by the lower box in (B) shows labeled cells lying in between the oculomotor nuclei, with dendrites extending between its subdivisions (red arrowheads). At the caudal end of III, where the caudal central subdivision (CC) is found, rabies-positive cells are present both ipsilaterally (F) and contralaterally (G). They stretch across the supraoculomotor area (SOA). Red arrows in (F, G) indicate multipolar neurons shown at higher magnification in (H, I), respectively. Scale bar for A and B is shown in C. Scale bar for E is shown in D. Scale bar for G is shown in F. Scale bar for I is shown H.

The pattern of labeled cells in the oculomotor region was the same at the 72- and 76-hour survival times, and we will use a 76-hour case for illustration. Most of the labeled rabies-positive cells were located in about the same locations at the rostral pole of III (Fig. 8A), with cells found in the ipsilateral EWpg. However, some cells were found more ventrally, on the contralateral side of the midline in SOA. At more caudal levels, the ipsilateral EWpg cells were joined by cells located in the contralateral EWpg and SOA (Figs. 8B, 8D, arrowheads). Note that the dendrites of the labeled cells in the left EWpg cross the midline (Fig. 8D). In addition, a small number of cells inhabited the area between the two oculomotor nuclei (Fig. 8B). These were bilaterally distributed, with an ipsilateral predominance, and their dendrites extended into the regions between the oculomotor subnuclei (Fig. 8D, arrowheads). At the caudal end of III, the pattern of labeling changed radically (Figs. 8F, 8G). Rabies-positive cells formed a broad band that crossed the SOA, bilaterally. These cells had medium-sized, multipolar somata (Figs. 8H, 8I). Their dendrites were sparsely branched and extended within the SOA.

**Figure 9:**
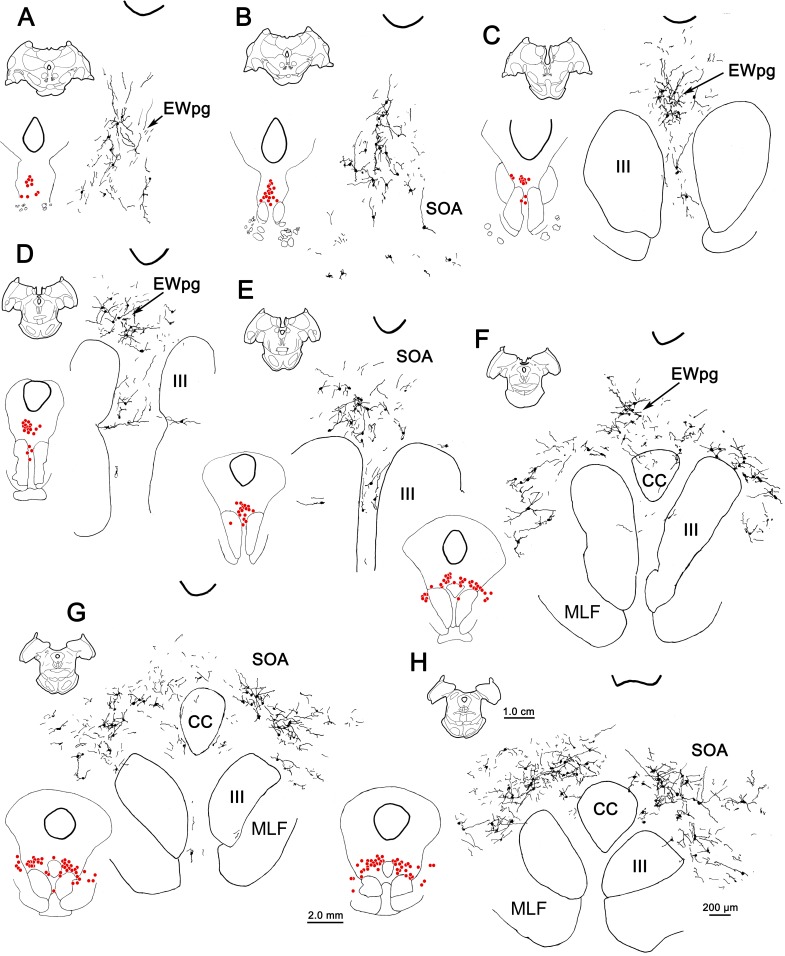
The distribution of labeled neurons in the preganglionic Edinger-Westphal nucleus (EWpg) and supraoculomotor area (SOA) at 76 hours after an injection of rabies into the left ciliary body (A–H). For each level, the section is shown, as well as a medium-magnification charting of the labeled cells as red dots. Rabies-positive cells were distributed from just rostral to the oculomotor nucleus (III) (A) to its caudal pole, where the caudal central (CC) subdivision is present (F–H) and the EWpg is no longer apparent (G, H). The labeled dendrites fill much of the supraoculomotor area (SOA).

**Figure 10:**
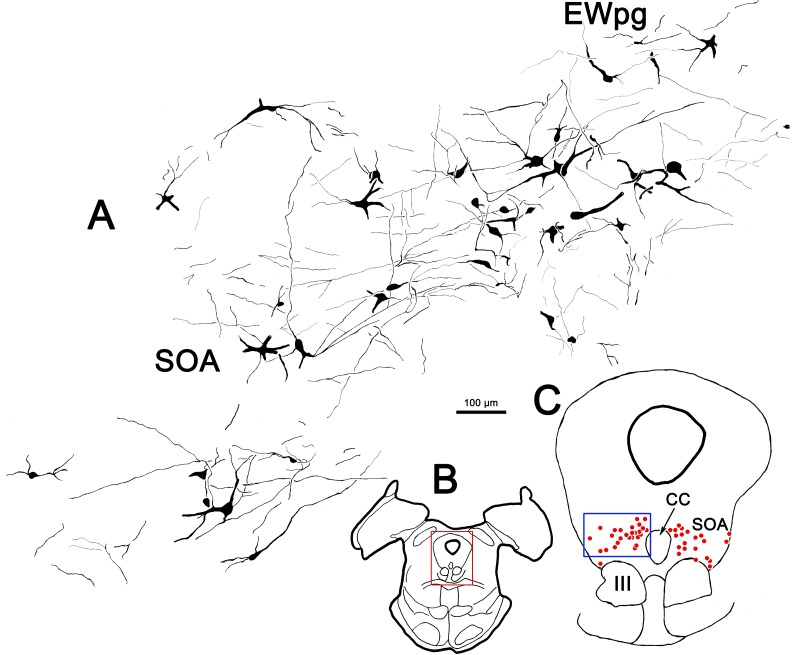
Illustration of the dendritic fields of rabies-positive cells in the ipsilateral supraoculomotor area (SOA). (B) The section illustrated is at the caudal end of the oculomotor nucleus (III). (C) Rabies-positive cells (red dots) labeled at 76 hours after the ciliary body injection were distributed in a bilateral band within SOA at this level. The region in the blue box is illustrated in (A). In (A), the dendrites of the labeled cells were largely constrained to the area occupied by the labeled cells in SOA and the preganglionic Edinger-Westphal nucleus (EWpg).

These features are further examined in the chartings of this case in Figure 9. Rostral to III (Fig. 9A), most of the cells were located in EWpg, ipsilateral to the injection. Their dendrites extended dorsally toward the cerebral aqueduct. Additional labeled cells were located ventral to EWpg, and these were distributed bilaterally. The same basic bilateral organization was observed at the rostral pole of III (Fig. 9B). Throughout the middle levels of III (Figs. 9C–E), most of the labeled cells were located within ipsilateral EWpg. However, other cells were located within the contralateral EWpg, and bilaterally in the SOA, ventral to EWpg. The dendrites of the labeled cells at these levels tend to occupy the midline swath where their cell bodies lie. A small group of cells was found bilaterally, in the region between the oculomotor nuclei, and some of these cells and their dendrites even appear to be located within the borders of III. It is possible that their location corresponds to narrow regions between the cytoarchitectonic subdivisions of III,^13^ where C-group cells have been described.^14^ At the caudal pole of III (Figs. 9F–H), the cells labeled with rabies were distributed as a bilateral band that filled the SOA and extended out into the MLF, lateral to III. This bilateral band became evident just rostral to the CC and it continued behind EWpg (Figs. 9G, 9H) until the caudal pole of III was reached. There, it abruptly ended. The organization of this caudal band is further illustrated in Figure 10, where the rabies-labeled cells on the ipsilateral side have been drawn at higher magnification. The dendrites of these multipolar cells covered considerable distances within the SOA, but rarely branched. The dendritic fields tended to extend mediolaterally and so were largely colocalized with the labeled somata.

**Figure 11:**
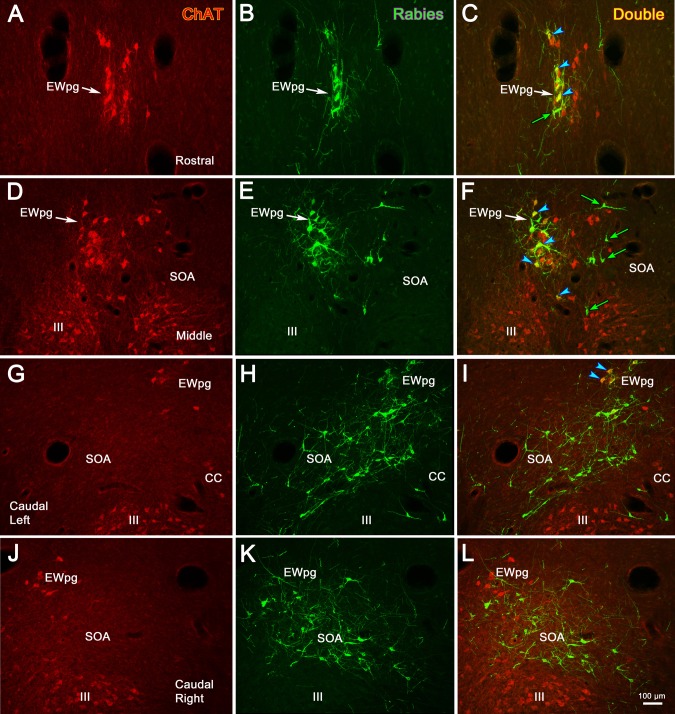
In animals that survived 76 hours after the injection of rabies into the left ciliary body, labeled premotor neurons are present. Examples from rostral to the oculomotor nucleus (III) (A–C), the middle of III (D–F), and the caudal end of III where the caudal central subdivision (CC) is present (G–I, ipsilateral; J–L, contralateral) are shown. Immunohistochemistry for choline acetyltransferase (ChAT) caused preganglionic motoneurons in the Edinger-Westphal nucleus (EWpg) and somatic motoneurons in III to fluoresce red (A, D, G, J). In contrast, the rabies-positive cells fluoresced green (B, E, H, K). The rabies-positive, ChAT-positive cells appear yellow (blue arrowheads) in the combined images (C, F, I). These double-labeled cells are confined to the ipsilateral EWpg. Numerous rabies-positive, ChAT-negative premotor neurons (green arrows) can be seen as green cells located bilaterally in the supraoculomotor area in the combined images (C, F, I, L). The scale for all plates is found in (L).

We again examined the transmitter content of the cells labeled by the trans-synaptic transport of rabies virus using the dual-immunofluorescence method in order to discriminate cholinergic motoneurons from the presumably noncholinergic, premotor population labeled at 76 hours after injection. Figure 11 shows the results of this analysis. At the rostral end of EWpg (Figs. 11A–C), most, but not all (Fig. 11C, green arrow), of the green fluorescing, rabies-labeled cells also fluoresced red, due to the presence of ChAT, and so appear yellow when the images are combined (Fig. 11C, blue arrowheads). At intermediate levels of III (Figs. 11D–F), the green fluorescing rabies-positive cells (Fig. 11E) were primarily found within the group of red fluorescing, ChAT-positive motoneurons of the EWpg, ipsilateral to the injection (Fig. 11D), and so were revealed as yellow cells when the images were combined (Fig. 11F, blue arrowheads). However, a number of green fluorescing rabies-positive cells were located on the contralateral side (Fig. 11E). These ChAT-negative, premotor neurons are indicated by green arrows (Fig. 11F). The distribution of premotor neurons at the level of the caudal central subdivision is shown for the ipsilateral (Figs. 11G–I) and contralateral (Figs. 11J–L) sides. Only a few of the rabies-positive cells were ChAT-positive motoneurons in the caudal pole of the EWpg (Fig. 11I, blue arrowheads). The vast majority of cells labeled by rabies at this level were ChAT-negative premotor neurons that fluoresced green. It should be noted that the rabies-positive cells found between the two oculomotor nuclei were not ChAT-positive, except for a few at the rostral pole of III (not illustrated).

**Figure 12:**
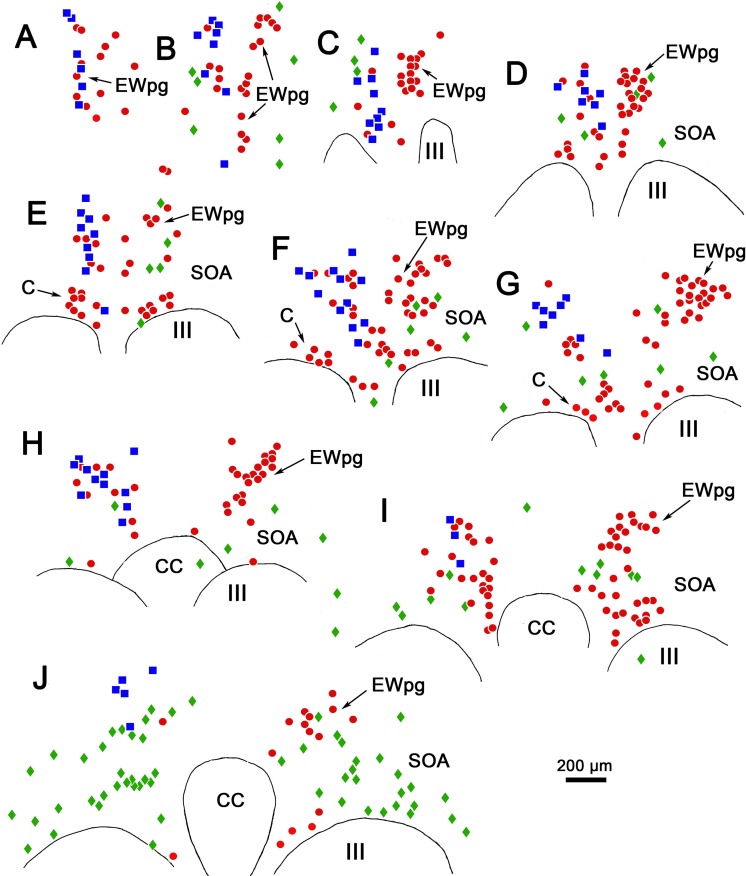
Distribution of rabies-positive premotor neurons labeled 76 hours after an injection into the left ciliary body. The cells that are just rabies-positive (green diamonds), just choline acetyltransferase (ChAT)-positive (red dots), and double labeled (blue squares) are found rostral to the oculomotor nucleus (A, B), in the supraoculomotor area (SOA) above the oculomotor nucleus (III) (C–J), and at the level of the caudal central subdivision (CC) (H–J). The ChAT-positive, rabies-negative motoneurons (red dots) are located bilaterally in the preganglionic Edinger-Westphal nucleus (EWpg) and in the C-group (C) (E–G). The ChAT-positive, rabies-positive preganglionic motoneurons (blue squares) are found in the ipsilateral EWpg. Rabies-positive, ChAT-negative premotor neurons (green diamonds) are present bilaterally. While they were present throughout the entire region (A–J), they are particularly prominent caudally (J), where they form a band in SOA.

The overall distribution of these populations is charted in Figure 12. On the side contralateral to the injection site (right side), the cholinergic motoneurons in EWpg are plotted as red dots (Figs. 12A–F). On the side ipsilateral to the injection site (left side), some of the ChAT-positive motoneurons in EWpg were not labeled with rabies (red dots), while many were double labeled; that is, they were rabies-positive, as well (blue squares). Other ChAT-positive, rabies-negative cells were located adjacent to the border of III. These presumably represent C-group motoneurons that supply multiply innervated muscle fibers. Premotor neurons that were rabies-positive but ChAT-negative (green diamonds) were also present. In the more rostral sections (Figs. 12A–G), these were relatively few in number and were mainly located on the contralateral side of the SOA. However, at the caudal end of the distribution (Fig. 12H), they became the dominant population, and extended laterally within the SOA on both sides of the midline.

## Discussion

Through the use of trans-synaptic transport of rabies virus, we have provided the first anatomic description of the premotor neurons that control lens accommodation. It is very likely that they correspond to the midbrain near response cells described electrophysiologically.^1,2,4,15,16^ These multipolar cells populate the SOA and have a bilateral distribution with respect to the eye they control. The number of cells is greatest at the level of the caudal pole of III. The results also demonstrate the presence of preganglionic motoneurons controlling lens accommodation throughout the rostrocaudal extent of a largely unitary EWpg.

### Interpretation of the Findings

Injection of the ciliary body in these experiments risks involvement of the vitreous and aqueous humor, and spread within the layers of the eye (Fig. 1). We did not see evidence of terminal or cellular label in the dorsal lateral geniculate that would indicate uptake from the vitreous by retinal ganglion cells. Similarly, the trigeminal ganglion (Fig. 2E) was free of somatic labeling and the trigeminal sensory nucleus lacked terminal and somatic labeling, indicating there was no uptake by somatosensory fibers supplying the eye. These results agree with previous characterizations of this virus as travelling solely in a retrograde fashion.^12,17,18^ It was certainly possible that some of the viral particles that ended up in the aqueous humor would have access to the sphincter or dilator pupillae muscle, as the aqueous humor bathes the iris. With respect to the parasympathetic innervation of the sphincter pupillae muscle, labeled cells were not observed in the olivary pretectal nucleus. Since olivary pretectal nucleus premotor neurons directly access pupillary preganglionic motoneurons in the EWpg,^19,20^ we conclude that there was no involvement of the pupillary sphincter. Similarly, no labeled postganglionic motoneurons in the superior cervical ganglion (Fig. 2C) or preganglionic motoneurons in the intermediolateral cell column of the upper thoracic spinal cord were labeled, precluding involvement of the pupillary dilator.

This negative finding with respect to the sympathetic system was somewhat surprising, since it has been suggested that the sympathetic system also provides input to the ciliary body that may influence lens accommodation,^21–23^ but see Richdale et al.^24^; and it certainly influences production of aqueous humor by the ciliary processes.^25–27^ Perhaps the uptake of virus by these sympathetic fibers was too rare an event to produce labeling. It was also possible that there might be uptake by parasympathetic fibers that control the perfusion of the retina. In mammals, these appear to utilize postganglionic motoneurons located in the pterygopalatine ganglion and preganglionic motoneurons located in the superior salivatory nuclei.^28^ However, we found no cellular labeling in either the ganglion (Fig. 2D) or this brainstem nucleus.

Another possible source of artifactual labeling of cells is spread to the extraocular muscles. A few labeled cells were observed within the borders of III, but we do not believe this represents uptake from extraocular muscles because it occurred only at later time points, indicating that these cells were premotor neurons, not motoneurons. There was also labeling of a few cells in the regions between the borders of the EWpg and III, and between the oculomotor nuclei at earlier time points (58 and 66 hours). C- and S-group motoneurons that supply multiply innervated extraocular muscle fibers and palisades are located in these areas.^29–34^ However, if these motoneurons were labeled, we would have expected that the premotoneurons in the abducens and prepositus nuclei would have been labeled at the later time points (>66 hours), since rabies virus placed in the distal lateral rectus muscle labels premotor inputs to abducens motoneurons at 60 hours.^18^ Since no such labeling was present, we believe these were preganglionic motoneurons located outside the borders of EWpg.

In analyzing the data, we must consider the time course of the labeling. At 58 hours, labeled cells were observed only ipsilaterally in EWpg, and most of the cells were quite lightly labeled, suggesting that the virus had been replicating in these second-order neurons for only a short period of time (Fig. 1). At 66 hours, the pattern of labeling was generally similar to that at the 58-hour time point, although all the EWpg neurons were now very densely labeled. However, at this point, a few lightly labeled non-motoneurons were observed within SOA and in other brainstem areas, suggesting transfer to third-order premotor neurons had begun. At later time points, a very different bilateral pattern of labeling was evident, indicating that premotor neurons were now fully involved in replication of the virus. Our interpretation of these data is that labeling of third-order premotor neurons had just begun at 66 hours and that it expanded until 76 hours. Indeed, there was evidence of light fourth-order labeling outside the perioculomotor region at 76 hours (not illustrated).

The presence of a few densely labeled cells at 58 hours may be due to direct preganglionic motoneuron projections to the scattered peptidergic and nitridergic ganglion cells located in the choroid,^35–37^ some of which are also present in the ciliary muscle.^38^ Earlier labeling of this preganglionic population may have led to the labeling of a small number of premotor neurons at 66 hours. It is also possible that the presence of this pathway explains the early findings that suggested a direct pathway to the ciliary muscle.^39^ However, it should be noted that there is currently no direct evidence that these cells are supplied by EWpg.^40,41^

### Preganglionic Motoneurons

This study represents the first anatomic description of identified lens accommodation-related preganglionic motoneurons in a primate. The labeled cells were present throughout the rostrocaudally running column of preganglionic motoneurons (Figs. 4–6). No areas of the nucleus were particularly devoid of labeled cells, which would have suggested the presence of a discrete pupil-related region. Previously, a trans-synaptic retrograde study in the cat had suggested that pupil-related preganglionic motoneurons are primarily located rostrally, in the anteromedian nucleus.^42^ However, the pupillary component of the monkey EWpg is very small,^43^ making a small pupillary region easy to overlook. Furthermore, substantial differences in the organization of EWpg in the feline and primate models make it very difficult to undertake meaningful comparisons between these species.^44,45^

The organization of the primate EWpg has been a matter of dispute. Some authorities indicate that this nucleus represents a unitary, rostrocaudally oriented cell column that lies dorsal to III and that extends into the anteromedian nucleus, rostral to III.^10,11,46^ Alternatively, it has been proposed that EWpg is organized into a set of separate subdivisions—the dorsal, medial, lateral, and accessory lateral visceral columns—plus two nuclei, the anteromedian and Perlia.^11,47^ It has been suggested that some of these subnuclei are specifically targeted by certain afferent inputs.^48,49^ In many ways, the present report reinforces the single column concept, as most of the labeled preganglionic motoneurons were arranged in a column above III. However, we also noted irregularities in the distribution: Some preganglionic motoneurons were located ventral to the borders of EWpg, and the nucleus itself varied from section to section (e.g., right and left nuclei in Figs. 6B, 12C). This may account for previous attempts to identify specific subdivisions, but the pattern of variation differs between animals and sections, making it inadvisable to identify specific subnuclei. Instead, the macaque EWpg is a fairly continuous group that encompasses the previously described^11^ anteromedian nucleus and the dorsal and lateral visceral columns. The motoneurons scattered in the SOA between EWpg and III would then be equivalent to the medial visceral column.^10^ We also saw a few preganglionic motoneurons between the oculomotor nuclei, an area sometimes defined as the nucleus of Perlia. Considering the lack of nuclear boundaries seen for the EWpg population of nonprimate mammals,^50^ it is not surprising that there is some scatter for the much better-organized preganglionic population of the macaque.

The fact that only a portion of the preganglionic motoneurons were infected by the rabies virus is of interest (Figs. 6, 12). The ciliary muscle requires equal degrees of action throughout the circumference of the lens for normal accommodation. Thus, one might predict that most, or even all, of the postganglionic motoneurons would receive input from each individual preganglionic cell. Our data suggest otherwise. The incomplete preganglionic labeling suggests that there is limited divergence, so individual postganglionic motoneurons may only receive input from a small number of preganglionic motoneurons. Intra-axonal staining of preganglionic fibers in the rabbit shows a similar constrained set of target cells.^51^ In this regard, the organization of the ciliary motoneurons is noteworthy. At the ultrastructural level, their somata are surrounded by a perisomatic neuropil that contains numerous synaptic contacts,^52–54^ but they also emit a few dendrites. Both these characteristics were evident in the virus-labeled postganglionic motoneurons (Figs. 2A, 2B). If the large dendrites represent a structural specialization for receiving more divergent preganglionic input, it still appears that these cells are dominated by this individual perisomatic input or that this is the main site of rabies transfer. If significant divergence does not occur at the level of the ganglion, the balanced activity in the muscle must be achieved by other means. One possibility, gap junctions between the ciliary muscle fibers,^55^ has been ruled out,^56^ so presumably central mechanisms must coordinate the activity of the ciliary muscle motor units in order to produce widespread, equivalent activation.

### Near Response Neurons

Mays^1^ reported the presence of cells in the midbrain with activity that encoded vergence angle and that did not encode conjugate gaze changes. He investigated cues that encode lens accommodation under monocular viewing conditions that separated vergence angle from target distance. This study revealed that some cells primarily encode vergence angle, some primarily encode accommodation changes, and others encode both. Judge and Cumming^2^ used a different testing setup and more directly measured lens accommodation. They found the same admixture of neurons whose firing was related to vergence angle, lens accommodation, or both. They electrically stimulated the region containing these cells and produced convergence and lens accommodation. It is presumed that these cells also encode activity related to pupillary diameter, the third leg of the near triad, but this has not been directly tested. Since the virus was injected into the ciliary muscle in the present study, it is reasonable to assume that we have labeled only the portion of the near response population that directs lens accommodation. However, as these investigators did not report any topographic differences between their subpopulations, it is reasonable to assume that the distribution of pure vergence neurons would be very similar.

Mays^1^ used microlesions to indicate the location of the recorded population and found that the cells lay immediately dorsal or lateral to III. Similarly, Das^16^ shows tracks and microlesions within the SOA where he found near response neurons, and Zhang and colleagues^4^ also placed their recorded units there. The location of these recorded units precisely matches the distribution of labeled premotor neurons we have found. It also overlaps with the distribution of premotor neurons labeled by rabies injections into the lateral rectus muscle of monkeys^18^ and the medial rectus muscle of guinea pigs,^9^ reinforcing the idea that the lens and vergence populations are intermixed. Indeed, the premotor population labeled from the lateral rectus muscle was concentrated caudally and bilaterally distributed like the population we illustrated here. This also agrees with the physiological findings indicating that divergence and convergence cells are intermixed.

Judge and Cumming^2^ described cells lying dorsal to III as well, but also reported near response cells at other locations. Mays and colleagues^3^ described neurons whose bursting activity was specifically related to the vergence velocity in the pretectum and in the midbrain reticular formation lateral to III. We have constrained the analysis contained in this paper to the population dorsal to III, but other premotor populations were labeled, and we will describe these neurons in future reports.

The discovery of midbrain near response cells, together with the description of neurons in the horizontal gaze center whose activity correlated with conjugate eye movements, supplied evidence for centers that fulfilled the theoretical specifications of Hering.^57^ He proposed that all eye movements could be specified by the interaction of a center for conjugate eye movement and a center for vergence eye movements. More recent reinvestigations of the pontine burst neurons^6,8^ indicated that in many cases the cells in the horizontal gaze center have activity that correlates primarily with the movement of just one of the eyes during a monocular eye movement when the new target distance differs from that of the fixation point. Based on this, it has been proposed that independent control of the eyes is utilized not just for disjunctive saccades, which are known to have different metrics than conjugate ones,^58^ but for all disjunctive eye movements. In this revision of the original monocular control theory proposal by von Helmholtz,^6,8,59^ the only exceptions are for symmetric eye movements due to target distance changes along the midline. It is proposed that these alone are controlled by the midbrain near response neurons.

The pathways that control the other components of the near response were not considered in monocular models.^59^ Each monocular controller could supply a separate signal for accommodation to EWpg, but there is little evidence for projection by the paramedian pontine reticular formation to EWpg, and we did not see any premotor neurons there (not illustrated). Alternatively, target distance information could be separately directed to the lens accommodation premotor neurons described here. If this is the case, it is curious that these cells seem to overlap so closely with near response premotor neurons supplying medial and lateral rectus motoneurons. The bilateral disposition of the premotor lens accommodation neurons does align with the suggestion that the near response population produces symmetric actions,^7^ although lens accommodation can be independently controlled.^60^ Furthermore, we noted that the dendrites of some EWpg cells extend across the midline to provide a morphologic substrate for binocular ciliary muscle activation. Of course, the present data do not prove that individual premotor neurons project bilaterally; intermixed ipsilaterally and contralaterally projecting populations could produce the bilateral distribution seen here.

The fact that the near response cells are particularly numerous in the caudal SOA must now be taken into account when considering possible afferents to this population. It has previously been proposed that the deep cerebellar nuclei target near response neurons.^61^ Examination of this work suggests that the terminals of the fastigial nucleus projection to SOA do have a caudal bias, but those of more lateral nuclei do not. The central mesencephalic reticular formation (cMRF) also supplies the SOA with input,^62^ and this terminal field is also denser caudally. It has recently been suggested that the superior colliculus may play a role in vergence eye movements^63,64^ and the superior colliculus is known to project to SOA,^65^ but whether the colliculus targets midbrain near response neurons is not known. It is our hope that this anatomic demonstration of the location of the midbrain near response neurons will lead to a greater understanding of the inputs that may govern the activity of these cells. In particular, we need to understand how target distance information that is distilled from blur and disparity signals in cortex gains access to the midbrain near response neuron population.
